# Technical Modifications of Double-J Stenting for Retroperitoneal Laparoscopic Dismembered Pyeloplasty in Children under 5 Years Old

**DOI:** 10.1371/journal.pone.0023073

**Published:** 2011-08-11

**Authors:** Zhi Chen, Xiang Chen, Yan-Cheng Luo

**Affiliations:** Department of Urology, Xiangya Hospital, Central South University, Changsha, Hunan, China; University of California, Merced, United States of America

## Abstract

Both antegrade stenting and retrograde stenting for retroperitoneal laparoscopic dismembered pyeloplasty in children have many disadvantages. In this work, we tried using an alternative technique of modified antegrade (MAG) double-J stenting for retroperitoneal laparoscopic dismembered pyeloplasty in children under 5 years old, analyzed our results using the conventional antegrade (CAG) and the MAG techniques of stent insertion for this procedure, and reported our experience with these techniques. Between December 2002 and July 2010, 77 children under 5 years old with ureteropelvic junction obstruction underwent retroperitoneal laparoscopic dismembered pyeloplasty. CAG and MAG double-J stenting were attempted, in the first 36 cases (mean age 27.1 months) and the following 41 cases (mean age 25.4 months), respectively. The stents were removed 4–6 weeks later via cystoscopy. Follow-up studies were performed with ultrasonography and intravenous urography at 3 and 12 months postoperatively. The results showed that successful stent placement without malpositioning was achieved in 31 of 36 (86%) and all 41 (100%) cases, in the CAG and MAG groups, respectively. The common factor of unsuccessful stent was the inability to across the ureterovesical junction. The mean stent insertion time was 10 min 54 s and 12 min 46 s in the CAG and MAG groups, respectively. The mean operating time was 176 min and 185 min in the CAG and MAG groups, respectively. No stent malpositioning occurred in the MAG group; in the CAG group, two children had a malpositioned stent in the distal ureter and one child presented with a severe hematuria. Twelve months follow-up showed no new onset of hydroureteronephrosis and hydronephrosis. Thus we concluded that the MAG double-J stenting seems more reliable than CAG stenting for retroperitoneal laparoscopic dismembered pyeloplasty in children under 5 years old, with greater success and lower complication rates.

## Introduction

Anderson–Hynes pyeloplasty is considered the gold standard for ureteropelvic junction obstruction (UPJO), proving its efficacy with a high success rate during long-term follow-up assessment [1], [2]. Since 1993 when laparoscopic pyeloplasty was initially reported by Kavoussi [3], it has proven to be a safe and effective procedure for UPJO, with a comparable success rate to that with the open technique [4], [5], [6], [7]. Furthermore, it has well-established advantages of laparoscopic surgery: less pain, shorter hospital stays, shorter convalescence, and less scarring [8].

Stent positioning is essential for minimizing complications from this procedure, particularly leakage, allowing more rapid improvement and resolution of hydronephrosis [9]. Various techniques of antegrade (AG) stenting for laparoscopic pyeloplasty have already been reported [10], [11], [12], [13], [14] and most surgeons prefer to place the stent AG during the anastomosis, as is used in open surgery usually as a blind insertion. Although some of these techniques are simple and efficient, there are still a certain number of stenting failures. The common factor for unsuccessful ureteral stenting is difficulty in negotiating the ureterovesical junction (UVJ) [15]. Furthermore, it is not always reliable to confirm that the distal end of the stent is positioned in the bladder and malpositioning of the stent often occurs [13].

Retrograde (RG) stenting has many disadvantages. Dissection and full mobilization may be more difficult owing to the decompression of the renal pelvis by the stent. The stent can be cut accidentally during dissection or laparoscopic manipulation might result in stent migration [16], [17], [18]. Furthermore, it might impede the identification of the extent of the stenosis and hinder trimming of the ureter and suturing of the posterior anastomosis [13], [19].

We performed conventional antegrade (CAG) stenting for retroperitoneal laparoscopic dismembered pyeloplasty in children in 2002. By July 2006, five failures had been encountered. To our surprise, these failures were all in patients under 5 years old and caused by inability to cross the UVJ. To achieve a greater success rate of double-J stenting in children under 5 years old, we tried using an alternative technique of modified antegrade (MAG) double-J stenting for retroperitoneal laparoscopic dismembered pyeloplasty. The current study aimed to analyze our results using the CAG and MAG techniques of stent insertion for retroperitoneal laparoscopic dismembered pyeloplasty in children under 5 years old and reported our experience with these techniques.

## Materials and Methods

We obtained approval for this study from the ethics committee at Xiangya Hospital, Central South University. Also, we obtained informed consent from the parents of all participants in our study. The informed consent was written and specified in the operative consent. Between December 2002 and July 2010, 77 consecutive children under 5 years old with UPJO (mean age 26.9 months, range 6–60) underwent retroperitoneal laparoscopic dismembered pyeloplasty by a single surgeon. Seventy-two had unilateral UPJO with a normal contralateral kidney and five had bilateral UPJO. Sixty-five children had symptomatic UPJO. Hydronephrosis was diagnosed antenatally in 12 children; the rest presented with pain (n = 21), urinary tract infection (n = 40), or abdominal distension (n = 4). All children underwent preoperative radiological imaging, including ultrasonography and diuretic renography, for the diagnosis of UPJO. The indications for surgery included an increasing degree of hydronephrosis, a low split renal function (<40%) and/or an obstructive pattern on diuretic renography and/or symptoms such as pain and urinary tract infection. None of the patients had undergone previous surgery. CAG and MAG double-J stenting were attempted in the first 36 cases (20 boys and 16 girls, mean age 27.1 months) and the following 41 cases (19 boys and 22 girls, mean age 25.4 months), respectively. Follow-up studies were performed with ultrasonography and intravenous urography at 3 and 12 months postoperatively.

### Retroperitoneal laparoscopic pyeloplasty technique

All procedures were performed with the patient in the lateral decubitus position under general anesthesia. Retroperitoneal laparoscopic pyeloplasty was routinely performed with four ports. A 1–1.5-cm transverse skin incision was made below the tip of the 12th rib. A small initial retroperitoneal space was created using an index finger. A 14-Fr rubber catheter attached to a midfinger of a latex glove was inserted into the initial retroperitoneal space, and the glove was filled with 300–500 mL gas and removed 3–5 min later. Under the guidance of the forefinger in the retroperitoneal space, a 5-mm trocar was made 2 cm above the anterior superior iliac spine for the camera and a 3-mm trocar was made approximately at the intersect of the anterior axillary line and subcostal margin of the 12th rib. After a 5-mm trocar was inserted into the retroperitoneal space through the initial skin incision, the incision was closed by two towel clamps, and then a pneumoretroperitoneum was established with the intra-retroperitoneal pressure of 8–10 mmHg. The fourth trocar (5 mm ) was made at the intersect of the midaxillary line and subcostal margin of the 11th rib. After careful dissection of the proximal ureter and pelvis, a dismembered Anderson–Hynes pyeloplasty was performed using 5-0 Vicryl interrupted sutures. Crossing vessels, when present, were carefully dissected free of the ureteropelvic junction (UPJ) and transposed when needed. A urethral catheter and a perinephric drain were placed routinely. The operative time was recorded from the placement of patients' position to the insertion of a urethral catheter.

### CAG stenting technique

A guidewire (or 3-Fr ureteral catheter) was inserted into the double-J stent (4.7 Fr 16 cm) through the first lateral port of its proximal end extracorporeally. The double-J stent with the guidewire was inserted into the ureter down to the bladder using laparoscopic forceps through the fourth trocar. After the proximal end of the stent was positioned in the renal pelvis, the guidewire was removed by grasping the stent using laparoscopic forceps to prevent its upward migration. The duration for stent insertion was recorded as the time from the entry of the stent to the port to the correct positioning of the stent in the pelvis. Cystoscopy was performed at the end if there were any concerns regarding positioning. A postoperative X-ray was not routinely performed.

### MAG stenting technique

Before the operation the patient was placed in the lithotomy position, a cystoscopy was performed and a 3-Fr ureteral catheter was inserted into the midureter and positioned with its tip a few centimeters below the UPJ. The following procedure was performed as described for retroperitoneal laparoscopic pyeloplasty technique. After suturing the reduced renal pelvis and the posterior wall with an interrupted suture, the ureteral catheter was gently grasped at its proximal end by an atraumatic forceps and extracorporeally introduced through the fourth port with its distal end exiting the external orifice of the urethra. The proximal end of this ureteral catheter was inserted into the lumen of a double-J stent (4.7 Fr, 13 cm for girls and 16 cm for boys) with a few centimeters inside, and both ends were sutured extracorporeally with 6-0 polydioxanone suture (PDS). The proximal end of the double-J stent was grasped by an atraumatic forceps to prevent its further downward migration, and the distal end of the ureteral catheter was pulled down until its proximal end exited the external orifice of the urethra, whereas the distal end of the double-J stent was pulled AG through the ureter and bladder and its proximal end was positioned at the pelviureteric anastomotic stoma. The PDS was cut and the ureteral catheter was removed.

At the end, the patient was placed in the lithotomy position again. The distal end of the double-J stent was pushed into the bladder by a cystoscope under direct vision in boys, or by a urethral catheter in girls. Postoperative X-ray was not routinely performed. The duration for stent insertion was recorded as the total time from entry of the stent to the port to securing the proximal loop of the stent into the renal pelvis and the time for RG placement and post-procedural cystoscopy (in boys). This time did not include the repositioning time.

## Results

Seventy-seven consecutive children under 5 years old underwent retroperitoneal laparoscopic dismembered pyeloplasty. The pre-, intra- and postoperative patient data are listed in Table 1. Crossing vessels were detected at surgery in seven children (three CAG and four MAG). No conversion to open procedures occurred in either group. In the CAG group, stents failed to cross the UVJ in three children and they received an alternate type of drainage (nephrostomy tube) during surgery. For three children Of the 33 successful stent insertions, the stent met with a little resistance and cystoscopy was performed at the end. Two children had a malpositioned lower end of the stent, which was retrieved at ureteroscopy and one had correct stenting positioning in the bladder. Thus, successful stent placement without malpositioning was achieved in 31 of 36 (86%) cases in the CAG group. In contrast, the MAG stenting was successful in all 41 children with no malpositioning. Also, there was no complications related to MAG stenting.

**Table 1:** Preoperative, intraoperative and postoperative data of patients.

	CAG group	MAG group
No. of patients: n	36	41
Boys :n	20	19
Girls :n	16	22
Mean age months (range)	27.1(7–60)	25.4(6–58)
Side:		
Left n (%)	19	16
Right n(%)	15	22
Bilateral n(%)	2	3
Primary procedure n	36	41
Crossing vessels: n	3	4
Mean stent insertion time (range)	10′54″(5′22″–24′19″)	12′46″(7′38″–16′41″)
Mean ureteral catheter insertion time (range)	No need	6′12(4′23″–7′45″)
Mean operative time (min): (range)	176(143–240)	185(137–249)
Conversions to open procedure: n	None	None
Stent correctly positioned: n (%)	31(86%)	41(100%)
Stent malpositioning: n (%)	2(5.4%)	None
Severe hematuria: n (%)	1(2.7%)	None

CAG = conventional antegrade MAG = modified antegrade.

The mean stent insertion time was 10 min 54 s (5 min 22 s to 24 min 19 s) and 12 min 46 s (7 min 38 s to 16 min 41 s), in the CAG and MAG groups, respectively. The mean operating time was 176 min (range 143 to 240) and 185 min (range 137 to 249), in the CAG and MAG groups, respectively. The mean time required for cystoscopy to place the ureteral catheter was 6 min 12 s (4 min 23 s to 7 min 45 s), as recorded from patient positioning to final ureteral catheter placement. The double-J stent was removed at 4–6 weeks. At 12-month follow-up, satisfactory drainage with decreased hydronephrosis on ultrasonography and intravenous urography was considered successful in all children. No new onset of hydroureteronephrosis was investigated.

## Discussion

Double-J stenting is essential for laparoscopic pyeloplasty, because it allows adequate urine drainage and prevents recurrent strictures during anastomotic healing. Our first AG stenting technique for retroperitoneal laparoscopic dismembered pyeloplasty in children was performed in 2002. By July 2006, five failures had been encountered. To our surprise, these failures were in patients younger than 5 years old. The common factor for unsuccessful ureteral stenting was difficulty in negotiating the UVJ. In response, we tried using an alternative technique, that is, MAG double-J stenting, in children under 5 years old. When we were convinced about the successful application of MAG stenting, we used it in all subsequent children under 5 years old. Although our experience has been split as regards the double-J stenting approach, we would like to emphasize that no strict randomization was done of the children into the CAG and MAG stenting groups, and surgical experience varied between the groups, which therefore were not comparable.

In the present study, the technique of the MAG double-J stenting combined the advantages of the AG and RG approaches. With the tip of the ureteral catheter a few centimeters below the UPJ, we preserved the advantages of AG stenting, including easier pelvic dissection without ureteric decompression caused by the stent [14], [15] and excision of the stenosed segment and redundant pelvis without a stent in the operative field [13], [14], [19], [20]. Furthermore, we avoided accidental cutting of the ureteral catheter during dissection [16]. Also, we preserved the advantages of RG stenting, including the possibility of excluding any other abnormality distal to UPJ obstruction [19], [21], [22]. Besides, identification of the ureter was eased by the presence of the ureteral catheter.

It is well known that it is not always reliable to confirm that the distal end of the stent is positioned in the bladder with blind AG insertion and malpositioning of the stent often occurs [13]. To ensure accurate positioning of the distal stent coil during AG stenting, various techniques have been described. Rodrigues et al [23] have suggested that the bladder should be filled with methylene blue, with its appearance through the stent holes as an indication of correct positioning. However, if reflux is present, the UVJ is probably incompetent and standard AG techniques should be less problematic, which makes concerns regarding false-positive methylene blue test results less valid. Fluoroscopy is also considered to confirm the stent position [24], especially after blind AG insertion. However, the routine use of fluoroscopy is limited because of its cost, availability, and radiation exposure [15]. In contrast, in our MAG technique, the distal end of the double-J stent was pushed into the bladder by a cystoscope under direct vision in boys (by a urethral catheter in girls). This is the significant advantage of MAG stenting that both the upper and lower ends of the stent are positioned visually and not by a blind insertion. Although that advantage is also present with RG stenting [15], it has the disadvantage that laparoscopic manipulation may result in stent migration upward during surgery [17], [18]. Thus, we were convinced that the distal stent coil was positioned properly and any chance of malpositioning was eliminated. Although various techniques of stenting, including AG and RG stenting, are still widely used in laparoscopic pyeloplasty, these techniques may not be able to eliminate the chance of stenting failure at all [10], [12], [14], [15], [19]. However, in our study, the successful stent placement in the MAG group was achieved in all patients, suggesting that our technique of MAG stenting seems more reliable.

Our technique has some similarities with that described by Wu et al [24]. In that study, the proximal end of the ureteral catheter was linked to the distal end of the double-J stent with a silk extracorporeally, whereas in our study these two ends were sutured directly with 6-0 PDS. However, fluoroscopy was routinely performed to check the double-J stent position in their study, which was not required in the present study because of accurate confirmation of the stent distal position with MAG stenting technique. Furthermore, Wu et al. [24] mainly focused on adults and our study involved children under 5 years of age. In addition, the introduction of the fourth trocar in our technique, which was not used in the study by Wu et al. [24], had several advantages. First, with laparoscopic scissors passing through this port into the retroperitoneal space, the incision of the renal pelvis and ureter could be facilitated for an identical direction of the ureter and the scissors. Second, the AG stent insertion through this port could also be easier to perform for the same reason. Third, passing other laparoscopic instruments through this port could help the surgeon to retract the tissue and obtain a good operative field.

With the proximal end of the ureteral catheter being inserted into the lumen of a double-J stent with a few centimeters, it was easier for the junction to negotiate the UVJ by withdrawing the ureteral catheter and the risk of UVJ trauma was decreased. Also, it was easier and faster to suture both ends of the ureteral catheter and the double-J stent extracorporeally. However, it was necessary to ensure that the junction of the ureteral catheter and the double-J stent was stable, to prevent segregation when the stent passed through the ureter and bladder.

In our study, it was considered an indication of successful stenting using blind CAG insertion that the stent passed through the ureter into the bladder smoothly and did not retract during the procedure. However, this criterion was not always reliable. In the CAG group, the stents in three children passed through the ureter with a little resistance and cystoscopy was performed essentially at the end of the surgical procedure. One child showed correct stenting positioning in the bladder but two other children showed a malpositioned lower end of the stent, which was retrieved via ureteroscopy.

The only disadvantage of the MAG stenting is that it requires additional cystoscopy (once in girls and twice in boys) for the placement of the ureteral catheter and pushing the distal end of the double-J into the bladder (in boys), which could increase the total operative time. However, in our series, the mean operative time in the MAG group was similar to that in the CAG group (185 *vs* 176 min ). This can be explained in part by the fact that although the children in the MAG group needed additional cystoscopy and repositioning the faster and more accurate confirmation of the stent distal position could contribute to decreasing the operative time. Furthermore, an easier identification of the ureter might also reduce the operative time. In contrast, whenever a CAG stent failed to go smoothly, more time was spent to confirm the stent distal position or repeated insertions were needed. In addition, if there were any concerns regarding positioning, cystoscopy was performed at the end of the procedure in the CAG group, which could also have extended the operative time.

It is worth noting that with a blind CAG approach, repeated insertions may result in ureteral injury thus probably leading to severe hematuria. In the CAG group, one child in whom the stent failed to cross the UVJ underwent repeated stent insertion on three occasions and presented with a severe postoperative hematuria. Thus, we considered that repeated stent insertion could only be done at most twice without any violence. As a result, we tried to use a ureteral catheter as a guide for the stent instead of a guidewire, to decrease ureteral injury during surgery. However, the risk of unsuccessful stenting can be increased accordingly because of the flexibility of the ureteral catheter.

Crossing vessels were encountered in seven children (three CAG and four MAG) in our study. Zhang et al. [20] have reported that prior ureteral stent placement could make it difficult to appreciate the influence of crossing vessels as possible causes of hydronephrosis, which could influence the choice of surgery. However, with the tip of the ureteral catheter a few centimeters below the UPJ, it is still easier to estimate the influence of crossing vessels. In our experience, crossing vessels, when present, are carefully dissected free of the UPJ and transposed if they can press the ureteropelvic anastomotic stoma after surgery.

In conclusion, the technique of MAG double-J stenting is efficient and easily reproducible with the combined advantages of the AG and RG approaches to eliminate risk of the stenting failure. In our experience, MAG double-J stenting seems more reliable than CAG stenting for this procedure in children under 5 years old, with greater success and lower complication rates.
